# The Conflict over Białowieża Forest in the Light of Philip Kitcher’s Ideal Deliberation

**DOI:** 10.1007/s00267-023-01906-w

**Published:** 2023-11-14

**Authors:** Ewa Kula, Zbigniew Wróblewski, Anna Starościc

**Affiliations:** The John Paul II Catholic University of Lublin, Al. Racławickie 14, 20-950, Lublin, Poland

**Keywords:** Environmental conflict, Forest protection, Białowieża Forest, Philip Kitcher, Deliberative democracy, Well-ordered science

## Abstract

Environmental conflicts involve many participants in the social life: citizens, foresters, the media, activists, politicians, officials and scientists. In this paper we pay special attention to scientists who provide the others with expert knowledge and proposals for solutions to ecological problems. Using the example of the ecological conflict around the strategy of protection of Białowieża Forest against the invasion of the spruce bark beetle (*Ips typographus*), we will present how these scientific functions were performed as well as what communication mistakes were made, and formulate a postulate to enrich forest management with a participatory model of social debate involving scientists representing all possible approaches to the problem. Our proposal applies Kitcher’s framework giving a ground for different stakeholders to come together to address complex environmental issues. Fitting into the trend of deliberative democracy, the paper provides an insight from philosophy that can be applied to controversial issues of policy and management, and how to influence an environmental change.

## Introduction

Various actors are involved in environmental conflicts such as social organisations and movements, state and local government institutions, local communities and business representatives. Most of them turn to expert knowledge as a fundamental factor in conflict resolution. In the light of this knowledge, the subject matter of the conflict, its causes and its potential effects as well as the ways in which it can be managed or resolved can also be identified. In the case of environmental conflicts, various aspects of the conflict may be visible to the layperson with the ‘naked eye’, e.g., the observable effects of an environmental disaster; whereas other aspects are only visible through the ‘armed eye’ of a scientist (expert), e.g. the anticipated negative effects or unintended side-effects of implementing some technical project. These effects become socially realised only through scientific knowledge, based on interpretations of cause-effect relationships not directly accessible to sensory cognition, e.g., a layperson is unable to ascertain a causal relationship between electromagnetic radiation and an increase in the incidence of cancer. Only scientific interpretation reveals the risks—we learn about them through the scientific arguments presented. The multifactorial determinants of potential dangers (complex causality generating environmental hazards) need scientific theory to bring out their mechanisms of action.

In order to be effectively implemented in social conflicts, the signalled role of scientific knowledge still requires at least three key conditions to be met. Firstly, it presupposes the condition of social trust in expert systems (scientists) as well as state and economic institutions funding science. Secondly, it assumes that the currently dominant model of scientific cognition (i.e., science) identifies and evaluates ecological problems adequately. Thirdly, universal access to knowledge is demanded, which also implies the need for broad-based—not narrowed to the educational system—education of the public (Kitcher 2011, especially Chapter 7: Applications and Access). Even a superficial analysis of several selected ecological conflicts makes it evident that, in practice, all these conditions can be contested (Czartoszewski 2003 esp. p. 9–21). External determinants of science in the form of sources of funding, science policy, as well as economic and social expectations place expert scientists on opposite sides of the same conflict. The internal determinants of science (the preferred model of science in a given field) foster a pluralism of scientific theories that provides ‘ammunition’ to the same extent for the different sides of the environmental conflict.

The conflict in the Białowieża Forest (hereinafter: BF), one of the most valuable ecological systems in Europe, is a salient example of the outlined situation of scientists’ participation in ecological conflicts, especially in the years 2016–2018, which we focus on in this article. This time period was chosen due to the fact that the conflict in question has acquired the dimension of an international ecological dispute. The dispute concerns the model for protecting BF during a period of increased pest invasion. Active protection of BF is proposed by some forest scientists and accepted by foresters, the local community and economic institutions whereas passive protection is advocated mostly by biologists and environmental organisations (Konczal 2016), both national (Workshop for All Beings, Polish Society for the Protection of Birds) as well as international ones (WWF, Greenpeace, Greenmind) (Strzyżyński 2015).

In democratic societies, diverse social conflicts should be resolved through debate and consensus. The aforementioned key elements of the social and professional ‘fabric of knowledge’, i.e., public trust in expert systems and their environment, acceptance of the dominant model of scientific cognition in a given field, and a common understanding of core scientific concepts and achievements, can be effectively strengthened through debate between all entities involved in the environmental conflict. The lesson to be learned from the referenced conflict shows that debate and consensus can be simulated or mishandled, and that a legal solution to the conflict may only lead to freeze it for a period of time (Wojajczyk 2021). In this article, we wish to outline a model of debate in which scientists may fulfil their role in relation to all parties involved and bring about a satisfactory consensus.

In this article, we use the debate model proposed by Philip Stuart Kitcher (Columbia University), one of the more influential living American philosophers of science (Couch and Pfeifer 2016, p. 1; Gonzalez 2011, pp. 7, 11). He develops the ideas of the American pragmatist John Dewey by adopting solutions to problems whose value can be measured by their practical utility. From this perspective, he analyses science and its role in society, attempting to reconcile the needs of researchers and the expectations of humankind at large in a view known as well-ordered science. In other words, he seeks balance between the values derived from the interests, knowledge and needs of scientists and the need to take into account the values expressed by the public (Couch and Pfeifer 2016; Kitcher 2012, pp. 200–204, 2015; Kaiser and Seide 2013, pp. 15–42).

## Methods

This paper is a systematic review of the selected literature on the proceedings of the BF conflict. The article consists of two main parts, i.e., a description of the environmental conflict and the actual deliberation as well as a presentation of Kitcher’s concept of science and the application of his model of debate to the conflict under analysis. The first part of study draws primarily on works relevant to forest management. Publicly available official documents and online sources are also consulted. The second part—more theoretical and normative—draws primarly on the works of Kitcher. Several secondary sources were also used, such as works in social philosophy or assessing Kitcher’s views. References to these sources are made through this paper.

### Białowieża Forest Dispute—A Factual Deliberation

BF is located in the north-eastern part of Poland. It is a forest area that includes the Białowieża National Park (BNP), established in 1932 as the second national park in Poland (restituted as Białowieża National Park in 1947) (Rada Ministrów 1947, 1996). This form of nature protection applies in Poland to areas with special natural, scientific and cultural values of more than 1,000 hectares, and aims to preserve biodiversity, restore deformed habitats and conduct scientific research in the protected area (Sejm RP 2004). The park area is covered by three forms of protection: strict, active and landscape one (Minister Środowiska 2014). The BNP covers 1/6 of the Polish part of Białowieża Forest, which is recognized as the best-preserved close-to-primeval natural forest in the European lowlands (Jaroszewicz et al. 2019). The strict protection area of BNP has been on the UNESCO World Heritage List since 1979. The territory covered by the UNESCO title was enlarged in 2014 and now covers the entire Polish part of the Białowieża Forest (Inscriptions on the World Heritage List 2023) and is a part of the World Network of Biosphere Reserves (for description and documents see: The Białowieża Forest World Heritage site 2023). The rest of the BF area is divided into 3 forest districts: Białowieża, Hajnówka and Browsk. They are managed by the State Forests National Forest Holding as part of the ‘Białowieża Forest’ Promotional Forest Complex, covering an area of approximately 52,000 ha. Since Poland’s accession to the European Union, the entire area of the Forest within the boundaries of the compact forest complex is covered by the Natura 2000 programme, whose main objective is to protect species and their habitats as well as natural habitats considered valuable on a European scale (Natura 2000—Environment—European Commission 2023). Moreover, in the area managed by the State Forests there are numerous nature reserves and monuments, also subject to protection (Janeczko and Parzych 2007). Thus, both national and EU nature conservation law apply to this area, which has been subject to many cultural transformations with documented origins dating back to medieval times (Samojlik et al. 2013). An additional difficulty in the BF conservation process is the fact that most of BF is located within Belarus, thus outside the European Union. The political frontier translates into the presence of blockades and fences in the area, which affects the migration of species and therefore also natural self-regulatory processes (Valasiuk et al. 2017).

Since the 1990s, the dispute over how to actively or passively protect BF has intensified. It focused mainly on the issue of whether the entire BF should be protected as a national park. Some scientists as well as foresters, employees of the State Forests company and local communities (both local people and local authorities representing them) were against this idea (Niedziałkowski 2016). On the other hand, another group of scientists focusing their research on various aspects of the functioning of the BF (mainly ecologists and biologists) began to raise the need for greater protection of the forest, especially its biodiversity (Blicharska and Angelstam 2010). These aspirations were supported by numerous environmental organizations as well as by the general public. After the decision in 1996 to enlarge the Białowieża National Park, subsequent projects concerned placing the entire forest under strict protection. The following years brought further discussion on how to protect the forest, which unfortunately did not bring any specific solutions (Blicharska and Van Herzele 2015). In this paper, we mainly focus on the period of 2016–2018 owing to the intensification of the dispute, which has acquired an international character due to much greater involvement of international organizations and institutions.

The origins of the analysed stage of the dispute are linked to the decision of the Minister of the Environment to increase logging in the Białowieża Forest District by 3 times, included in an annex of March 2016 to the Forest Management Plan of the Białowieża Forest District drawn up the years 2012–2021 (Decyzja Ministra Środowiska Zatwierdzająca Aneks Do Planu Urządzenia Lasu Sporządzonego Na Lata 2012–2021 Dla Nadleśnictwa Białowieża 2016). It was connected to the frequently occurring gradations of spruce bark beetle (*Ips typographus*) reported by forest districts at the territory of BF, for whose main host plant is the Norway spruce (*Picea abies*—about 25% of the share in BF stands) (Aneks Do Opinii Rady Naukowej Leśnictwa Przy Prezesie Rady Ministrów Rzeczpospolitej Polskiej w Sprawie Zamierania Drzewostanów Świerkowych Na Obszarze Nadleśnictw Białowieża, Browsk i Hajnówka Wchodzących w Skład Leśnego Kompleksu Promocyjnego “Puszcza Białowieska” 2016). The removal of dry and spruce bark beetle-infested trees was intended to reduce the population of the beetle and to protect the high level of diversity and natural richness of forest ecosystems under active nature conservation principles (Grodzki 2016). On the basis of the annex, a decision was issued in February 2017 by the Director General of the State Forests to remove trees infested by the bark beetle and to harvest trees posing a threat to public safety and fire hazards in all age classes of stands in the Białowieża, Browsk and Hajnówka Forest Districts. Logging with the use of heavy equipment commenced in April 2017.

In May 2017, environmentalists’ protests and logging blockades began (one of the first was a protest organised by Greenpeace activists and the Wild Poland Foundation in Czerlonka Leśna in the Hajnówka municipality). The issue was taken up by the national and then international media, reporting that the amount of timber harvested was the highest in ten years (National Geographic Polska 2017). The largest number of trees was cut down in the Hajnówka Forest District, the smallest in the Białowieża Forest District. Due to increasing public protests, on 20 July 2017 the European Commission applied to the Court of Justice of the EU (CJEU) against the Republic of Poland (European Commission 2017), arguing that the ongoing logging threatens the integrity of the Natura 2000 site and thus constitutes a breach of an EU Member State’s obligation (Complaint to the European Commission Concerning Alleged Breach of Union Law; Failure to Comply with Articles 6(2) and 6(3) of the Habitats Directive and Article 4(4) of the Birds Directive (by Virtue of Article 7 of the Habitats Directive) in Relation to the Revised Forest Management Plan for Bialowieza Forest District 2016). On 27 July 2017 the Court decided to order an immediate halt to the logging until the parties had been heard and the dispute resolved. Protests conducted by supporters of the cessation of logging intensified in August 2017 due to the ongoing dispute in the media.

The local community constituted an essential group involved in the dispute. Surveys conducted in the BF area even before this stage of the dispute showed that local residents were not in favour of extending the strict protection area, as their livelihood depended on the use of natural resources (wood harvested as fuel and raw material for local businesses) (Blicharska and Van Herzele 2015). In their minds, foresters belong to the local community and enjoy great prestige (Niedziałkowski 2016), while environmentalists and scientists are regarded as outsiders with no understanding of their way of life (Blicharska and Angelstam 2010). Some locals, such as those living from tourism e.g. related to birdwatching, considered the benefits of stopping logging and expanding the strict protection area (Czeszczewik et al. 2019).

On 11 September 2017 the arguments put forward by the attorneys of the European Commission as applicant and the representatives of the Government of the Republic of Poland as defendant were heard before the CJEU in Luxembourg. After reviewing the documentation presented, the Court decided in November 2017 to order the cessation of logging under threat of financial penalties (these were set at €100,000 per day) (Poland Must Immediately Cease Its Active Forest Management Operations in the Białowieża Forest, except in Exceptional Cases Where They Are Strictly Necessary to Ensure Public Safety 2017), followed by a hearing held on 12 December 2017. In January 2018, there was a change in the position of Minister of Environment in the Polish government—the previous minister was one of the main figures involved in the media dispute.

On 17 April 2018 the Court of Justice of the EU ruled that the logging was contrary to the EU law. It found the following breaches: adopting an annex to the forest management plan of the Białowieża Forest District without ensuring that it would not adversely affect the integrity of the site of EU interest; failing to ensure the protection measures required under the Birds Directive and the bird species included therein; and failing to ensure the protection of species included under the provisions of the Habitats Directive (European Commission 2018).

This decision brought to an end one of the most heated stages of the dispute over how to protect Białowieża Forest, one of the noisiest environmental disputes in Poland in recent years^1^. The scientific community played a very essential role in this dispute. The nature of BF is the object of research by numerous scientific teams (to name just few: Scientific Laboratory of the BNP, European Centre for Natural Forests of the Forest Research Institute, Białowieża Geobotanical Station of the University of Warsaw, institutes of the Polish Academy of Sciences), as well as individual researchers. Their interests are focused on the fauna of BF as well as plant communities, especially forest communities (Jaroszewicz et al. 2019). Interesting research was also conducted on rare species of fungi (Karasiński et al. 2009). Decisions taken by the Minister of the Environment in the Polish government related to increased logging and the removal of diseased spruce trees were based on expert opinions of forest science specialists, mainly affiliated to the Forest Research Institute. The Minister of Environment Jan Szyszko himself also represented this field of science, being a professor of forest sciences. Before the CJEU, he was substantively supported by the Director General of the State Forests, a graduate of the Faculty of Forestry of the Warsaw University of Life Sciences^2^. The non-governmental environmental organisations protesting against logging, on the other hand, referred to the research of specialists in biological sciences (Kujawa et al. 2016).

The discussion regarding forms of nature conservation in BF has been ongoing for many years between proponents of active and passive protection of Białowieża Forest. Advocates of active protection believe that the unique forest located within the area of BF and its biodiversity can only be protected by applying conservation measures. They emphasise the fact that without existing protection measures, it would be impossible to restore BF forest stand to its current state (Szwagrzyk 2016). They also argue that the BF area is too small to leave nature undisturbed in order to sufficiently protect its biodiversity and other values that we associate with protected areas (Brzeziecki et al. 2017, 2018). Those in favour of passive protection advocate the complete and permanent abandonment of direct human interference in the state of ecosystems, creations and components of nature and the establishment of a national park on the entire Polish side of BF (Blicharska and Angelstam 2010; Blicharska and Van Herzele 2015). In their view, dead and decaying trees do not mean the destruction of BF, but are important for other species inhabiting the area (Czeszczewik et al. 2013; Mikusiński et al. 2018). Conducting silvicultural techniques in commercial forests results in the irreversible transformation of further parts of BF, which loses its value as a unique ecosystem (Kujawa et al. 2016).

These two ways of thinking are also apparent in the approach to the spruce bark beetle gradation, which constituted the basis of this phase of the BF dispute. Foresters intended to control it by removing infested trees to protect the compactness of the stand (Hilszczański and Starzyk 2017). They insisted that this was the only way to protect BF (Grodzki 2016), especially given the drought and global warming (Boczoń et al. 2018). Naturalists, on the other hand, protested against this practice by postulating that natural self-regulatory processes would manage the problem (Wesołowski et al. 2016, 2018, 2019). Thus, the dispute between naturalists and foresters over how Białowieża Forest should be managed is part of a global dispute over conservation goals and methods, which in turn is a dispute over values (Witkowski 2017). To be clear, we consciously idealized the types of groups, contrasting two of them: ecologists and foresters, summarizing the conflict over how to protect the BF. The actual conflict had not only a scientific aspect, but also an ideological, political, and economic one (Wesołowski et al. 2016). This simplification more efficiently introduces the proposed model for resolving social conflicts in subsequent parts of the paper.

### The Concept of Well-ordered Science—Ideal Deliberation

Philip Kitcher points to the social context of practising science, which in his work results in addressing the issues of guiding scientific cognition, building promising research strategies and defining the role of scientists in the perspective of human-wide needs (Kitcher 2001, 2011; Kitcher and Keller 2017). Hence, the formulation of the ideal of well-ordered science is undertaken. This ideal is based on the conviction that the scientific inquiry is aimed to discover significant truths, captured both cognitively and practically, which is vital in terms of the procedures for selecting research problems (Kitcher 2011; Kitcher and Barker 2014, p. 137). The significance of the questions addressed by science corresponds to the needs of people and serves goals that are important from the point of view of society (with a view to satisfying these goals and, as a result, the successful development of humankind). The practice of science is understood here as directing the collective cognitive effort towards a mutually shared goal, which in the long run is aimed to foster the improvement of the human cognitive situation (meliorism) (cf. James 1987, p. 509). The recognition that science is not a value-free sphere, which manifests itself e.g., in the choice of research objectives and the planning of scientific research is an essential element of this concept. In addition, an important place is given to the human being as part of a wider whole, i.e., society that strives by joint efforts to solve various problems affecting it.

Kitcher himself identifies the following conditions for well-ordered science (cf. Kitcher 2011, pp. 105–137; Kitcher and Barker 2014, p. 151):

1. The lines of research that should be undertaken find favour through ideal deliberation.
2. The ways of conducting this research should are consistent with the standards accepted by the ideal deliberation.
3. Decisions concerning the issue of what results to include in the evolving set of accepted scientific claims have found acceptance in the ideal deliberation.
4. The practical application of scientific knowledge has found support through ideal deliberation.

Polemic and consensus, realised in a key-category: ideal deliberation is an essential element of this model practice of science. Aware of the possibility of conflicts between significant truth and other socially desirable values, Kitcher engages this type of deliberation in order to assess the proper functioning of scientific research, especially from the perspective of promoting collective values. It provides an alternative to the democratic will of the majority, which leads to the ‘tyranny of ignorance’ (Kitcher 2011, p. 118). The broadly defined medical industry is the ground on which the need for the application of the ideal of well-ordered science resounds. Kitcher points out (Flory and Kitcher 2004; Kitcher and Barker 2014; Reiss and Kitcher 2010, pp. 153–154) that funds allocated to the reduction of infectious diseases affecting predominantly poor communities are far lower than in the case of the cosmetics industry, whose recipients are affluent societies. Despite noticeable advances in medicine, there are still a number of ‘forgotten diseases’ that affect millions of people in poor parts of the world. The prevention and treatment of neglected tropical diseases (NTDs) is one example of an unresolved problem (cf. WHO 2020). Kitcher and Barker (Kitcher and Barker 2014) suggest that the ideal of well-ordered science would encourage the addressing of non-commercial problems, thereby valuing problems relevant to human progress. Kitcher’s views are formulated against the thesis that it is the scientific community that has the best insight into what common goods are to be satisfied by scientific research. They are summarised by an anecdote about scientists who have come to African pastoralists with an offer to develop a vaccine to protect their children. The pastoralists ask for time to consider the offer and then come with the suggestion that, from their point of view, a vaccine for the goats they raise is highly more preferable to a vaccine for their children (Kitcher 2011, pp. 118–119). No less important, however, is the possibility of conducting basic research, which—without expert judgement—could often be described as useless (just as it was difficult to predict that Thomas H. Morgan’s research on the fruit fly would play a considerable role in heredity research), or ventures driven by the satisfaction of cognitive curiosity. Thus, Kitcher emphasises the importance of translating ‘expert knowledge’ into ‘knowledge for all’ (Kitcher 2011, p. 111; Kitcher and Barker 2014, p. 154).

The ideal deliberation, which constitutes an essential element of well-ordered science, is defined by the following postulates: (1) the discussion on recognised values must take place within the entire human species, taking into consideration the requirement of responsibility for future generations; (2) the discussion must be held in mutual respect and engagement; (3) throughout the process under discussion, a situation is sought in which every person has serious equal opportunities to achieve a worthwhile life (Kitcher 2011, pp. 50, 57). At the same time, Kitcher stipulates that discussion participants must not resort to false beliefs about the world and should skilfully recognise the consequences of the actions and arrangements under discussion for all people, as well as at the same time reveal their expectations both at the starting point of the discussion and in the course of it. These conditions are essential in order to exclude voices that disrupt the ideal deliberation, ensuring the maximum objectivity and reasonableness of the discourse.

These characteristics make the ideal debate a platform for shaping needs in solidarity by taking into consideration the whole spectrum of both possible and rational demands, and its process—as already mentioned—is ultimately determined by the need to reach a consensus. The selection of the relevant problems and the direction of their solution is dictated by how important the issues to be addressed are for the entire human community, especially since representatives of various backgrounds and options are expected to participate in the discussion. Such designed ideal deliberation is accompanied by the realisation that society is diverse in terms of the knowledge and abilities of its members, and, at the same time, a great value is placed on the freedom of individuals understood as the possibility to shape their own lives. Here an individual is part of a wider whole—a society that strives together to solve the problems it faces. Thus, well-ordered science—embedded in the building of a research community—is such when “its specification of the problems to be pursued would be endorsed by an ideal conception, embodying all human points of view, under conditions of mutual engagement” (Kitcher 2011, p. 106).

This approach is highly participatory. The need to focus all possible points of view in this ideal deliberation is to be secured by the requirements Kitcher places on the participating deliberators and the conditions (stages) he indicates for reaching consensus. First and foremost, the deliberators should represent all possible attitudes, be knowledgeable about the state of research and see the prospects for its development as well as understand the needs of others, including minorities. It is of particular importance that deliberators have an open and non-dogmatic attitude. When participating in a debate, they should take into consideration the expectations and aspirations of other participants in the ideal deliberation (mutual engagement) and (subjectively and objectively) new information on the problem under discussion (Kitcher 2011, pp. 114–115; cf. Kawalec 2015, pp. 468–470).

Two aspects of the ideal deliberation are worth noting in terms of its practical application. The first is the size of the deliberative group. Kitcher prefers discussions as large as possible in terms of participants and viewpoints, appreciating the needs of the entire human population, including future generations. For practical reasons, he encourages the ideal deliberation to be held in smaller communities. The second is the key role of experts and their scientific authority in the ideal deliberation process. They act in two roles—both as tutors, relaying knowledge of the current state of scientific research to the other participants in the deliberation, as well as being deliberators—active representatives of their social group (Kitcher 2011, p. 124).

And even if the arguments for Kitcher’s position were clear, there would be the issue of participation which “is not just a matter of representing people, but of the ideas and values which they carry with them” (Bulkeley and Mol 2003, p. 151). These practical aspects of Kitcher’s philosophy provide an opportunity to test it with a specific example of a conflict in which scientists play an essential role.

### Postulates—Factual Deliberation Vs Ideal Deliberation

An analysis of the dispute surrounding BF leads to the conclusion that the parties involved in the conflict were unable to reach each other and failed to undertake due effort towards mutual understanding. This observation applies to all participants in the dispute, including representatives of the scientific community—in simple terms, foresters and biologists. In spite of attempts to present their arguments, the scientific community, even if only in the form of scientific articles, failed to find common ground for discourse as well as to engage in a reliable polemic (Hilszczański and Jaworski 2018; Wesołowski et al. 2019), which consequently turned into an argument of international reach. It is noted that participants in the dispute were guided by different values, some of which overlapped (especially the desire to preserve biodiversity in BF area) and some of which conflicted with each other (protection of unique plant and animal species, naturalness as an ecological value and heritage for future generations on the one hand, and cultural values, tradition and socio-economic values on the other). What is understood as different value systems: scientists face problems, theories, solutions and their applications, make choices, compare, evaluate and decide whether they are progressive or not, and their decisions are influenced by cognitive values, but also other determinants including non-cognitive ones, in which these values are rooted. Thus, we argue that this is a particularly difficult dispute, as it is rooted in different value systems, which in a broader perspective also gives rise to conflicts of interest (cf. Blicharska et al. 2020). An attempt to resolve the impasse would be to apply the assumptions of Philip Kitcher’s participatory concept of well-ordered science with its idea of discovering significant truths through ‘ideal deliberation’. Which truths are significant have nothing to do with semantics but is context-dependent and focused on issues that matter to people. Possible conflicts between significant truths and socially significant values must be taken into account (Kitcher 2001, p. 110).

The ideal deliberation is a manifestation of influential deliberative democracy or discursive democracy (Bächtiger et al. 2018, esp. chapter Antonio Floridia, The Origins of the Deliberative Turn). In political philosophy, deliberative democracy is a form of legitimation of power and public order, where public dialogue is expected to address the crisis of legitimation of power and communicative morality to ensure the requirement of universality (Rawls 1994, 2012; Habermas, 1999, 2002, 2005; Gutmann and Thompson 2004, 2006b, 2006a; Ricoeur 2003; Bächtiger et al. 2018, esp. chapter Simone Chambers, The Philosophic Origins of Deliberative Ideals). In this vein, Kitcher draws attention to the social dimension of scientific cognition, emphasising the need to build a research community beyond the scientific community. Based on the requirements he puts on the participating deliberators in an ideal deliberation, and the conditions (stages) he indicates for reaching consensus, we design a possible way of guiding the Białowieża dispute. Since Kitcher fails to provide specific guidance on deliberation design (Kitcher 2021), we supplement his ideal with our own suggestions.

The location of the debate and the selection of participants would be the first element of the ideal deliberation designed here. At the starting point it is proposed to select a setting in which the ideal deliberation can be held. On home ground, university professors are held in considerable public esteem (Centre for Social Opinion Research 2019), hence a university seems a convenient place to hold it, and its professors could direct the debate as moderators. It seems that philosophers would particularly be best placed in this role as a group which, being wisdom-lovers (Plato 1973; Verovšek 2021)—has a particular predisposition to argue (Brock 1987; Wojtysiak 2015): they have a distinctive methodological and axiological awareness; they are concerned with accuracy and criticism; they know how to make the problems they take up as the subject of discussion as rational as possible; they creatively argue and refute arguments; they are distinguished by their ability to systematise data from both common sense and the sciences; they have desirable discussion techniques such as identifying errors peculiar to the verbal expression of ideas. In addition to the aforementioned ‘technical’ competences, philosophers can contribute a ‘bird’s eye’ perspective to the discussion of complex issues in which axiological, empirical and conceptual problems are intermingled. The structuring of these issues, the isolation of the different types of problems, the identification of the fields of conflict, the definition of the values involved in the dispute—these are tasks that philosophers can effectively undertake. The above characteristics are indirectly confirmed by Kitcher when he calls guiding philosophers experts, i.e. “people who have particular knowledge or skills that are pertinent to some issue, that they can employ to guide discussion of that issue”. Being philosophical midwifery, they would be those who “can help others pose the questions clearly and sharply, who can introduce resources from the history of philosophy, who can expose difficulties in combining various positions, and so on” (Kitcher 2013a, p. 203). Additionally, the participation of philosophers seems justified insofar as it fits in with Kitcher’s postulated idea of a reconstruction of philosophy aimed at renewing its ties to everyday life, even as a ‘return of philosophy to life (Dewey 1954; Kitcher 2023, 2013b, 2012, pp. xv–xvi, 2011; Starościc, 2015). The distinction of the scientific community should not suggest its superiority but is convinient on a practice.

Participating in an ideal deliberation the deliberators should represent all possible stances essential to the dispute, have knowledge of the state of research and perceive its prospects for development, as well as understand the needs of others (cf. Bohman 2006). We treat the dispute over how to protect BF—somewhat arbitrarily—as a scientific dispute; nevertheless, following Kitcher’s proposal, we propose to include a number of stakeholders from outside the scientific community who have their own expectations regarding the formulated problems and their solutions, or representatives of those groups to be affected by these decisions (future generations). These measures are aimed at designing the debate that is possibly the most independent of either side of the conflict. The essence of the model of well-ordered science is to present all opinions and arguments on a given issue, to listen to the rationale of the participating parties and to be ready to change the position if there is reason to do so (participants do not insist on their position). As consensus is reached through the exchange of arguments, it is assumed that the participants in the deliberation are able to demonstrate the rationale for their beliefs. It is also assumed that the participants are well informed about the issue, the state of knowledge or the consequences of previous activities in the field. Participants are not required to bring new knowledge to the discussion, but above all to confront their opinions, preferences, principles or values with scientific data (cf. Pellizzoni 2003). This can be helped by information material dedicated to current BF issues.

The substantive preparation of the participants in the debate may face accusations of bias (who and what information has been included in the background material?). For this reason, we propose to structure the debate in two levels, where the first level is purely expert (academic) and the second one is of a general nature.

It is suggested that the first level of the deliberation serve to identify the areas which the problem encompasses, providing the basis for information material on the BF conflict. Its participants would represent both proponents of active and passive conservation, which—in a nutshell—corresponds to representatives of forest and biological sciences as well as experts from disciplines such as sociology, economics and finance, management, political science, law, earth and environmental sciences, environmental engineering, socio-economic geography and spatial management. It is assumed that, also at this stage, experts will address the successes and failures of the various means of solving analogous problems by revealing the objectives of the measures adopted (Kitcher 2011, p. 120). Most interestingly, attempts to reveal the motivations behind the expressed proposals for action on BF have already been made. E.g., in 2010–2011 individual in-depth interviews were conducted with 36 people representing different social groups involved in the dispute so as to investigate the reasons for opposing an enlarging the area of the national park. These clearly identified a division between two ‘coalitions’: forestry and conservation (Niedziałkowski 2016). A group of researchers who identified 20 scientists—BF specialists (10 so-called experts and 10 facilitators) in 2017–2018 provided a noteworthy assessment of the conflict over BF. They primarily highlighted the conflicting values and priorities among BF stakeholder groups that result in a lack of mutual trust. They concluded that evidence-based knowledge alone is insufficient to manage this type of conflict. It is essential to manage the conflict through a body recognised by all groups, which will arrange for all groups involved to participate in the decision-making process. As a first step of a potential debate, they agreed to prepare a publication that involved both sides of the conflict—a collaboration to generate new evidence (Blicharska et al. 2020).

The second level of the deliberation would implement Kitcher’s requirement of universality (Kitcher 2011, pp. 115–118; Kitcher and Barker 2014, pp. 136–139, 150–151), i.e., the deliberation participants would represent all possible viewpoints interested in resolving the Białowieża dispute, including the experts participating in the first level debate. All points of view relevant to the case should be taken into account. The model does not exclude any stakeholder group or researchers. Selection of participants for the debate is determined to the type of problem the community is facing. Deficiencies in representation can only be explained by the human factors (negligence or lack of interest). Participants of this stage are tutored on the basis of the materials developed at level one of the deliberation. It is proposed that the briefing materials are prepared in both cases by the moderators of the deliberation—by philosophers. The assumption is that, as human beings, we should be aware of our limitations, especially in terms of the knowledge we possess, and thus should draw on the knowledge of others if we wish our intentions to become a reality.

The course of an ideal deliberation at the two levels outlined would correspond to Kitcher’s distinguished stages of consensus building. These refer to the following activities (Kitcher 2011, pp. 466–469; cf. Kawalec 2015):

(1) Clarification of the problem: Participants in the deliberation obtain information concerning the issue and its context (Kitcher 2011, p. 114). Both the cognitive and practical aspects of the relevance of the approaches revealed in the dispute are essential. Deliberators present their view of the matter. In the second stage, as indicated above, participants are provided with materials, as objectivised as possible, to familiarise themselves with the objectives of the respective approaches, the results achieved to date and the associated opportunities in the matter. The ‘technical’ issues are explained by experts in the field, who justify the relevance of certain research, developments or unsolved problems and suggest whether undertaking them will result in practical solutions or perhaps deepen the understanding of an issue.

This stage is intended to deepen knowledge of the issue. It is assumed that, in the light of the information obtained, the participants in the dispute will revise their initial preferences taking into account the emerging information regarding the issue, including taking into account the (cognitive and non-cognitive) values that condition certain positions. The role of philosophers would amount to, inter alia, identifying the values that guide the parties involved in the dispute, attempting to combine the revealed preferences to obtain the values sought, or attempting to find common ground between the various values.

(2) Evaluation: Deliberators participate in a discussion in which they would present their positions, determine the significance of specific issues and preferred values, while providing factual justification. Depending on the number of participants, an ideal deliberation can commence by discussing the issue in small groups with the ‘technical’ participation of philosophers. With the assistance of experts in a given field, the participants confront their beliefs about the issue under consideration and adjust them based on the judgements of the other participants in the discourse (taking into consideration the consequences of possible decisions, their implications for society, for the inhabitants of different parts of the world and for future generations). With a small group of participants (under 30 people), allowing direct dialogue, the division into working groups can be omitted. This stage continues until a situation is reached in which each participant is satisfied. Due to the limitations of this paper, we have omitted details for ideal deliberation. At the same time, we believe that one of the most important advantages of this proposal is the attempt to involve all stakeholders in the decision-making process. During deliberation, we could learn the opinions of all parties to the conflict and at the same time be able to distinguish the arguments behind these opinions (Elster 1998).

(3) Selection: Aware of the assumptions behind certain positions and anticipating the consequences of possible decisions, deliberators formulate a common conclusion. Given the opportunity to draw on expert opinion in assessing the success of a given endeavour or outcome, deliberators select an agreed-upon method of resolving a disputed issue. The solution reached could take into account “the satisfaction of cognitive curiosity, the framework of practical interference, long-term benefits and immediate consequences” (Kitcher 2001, p. 119). The example of the only Model Forest, created on 15 September 2015 at the premises of the Oborniki Forest District, is an interesting illustration of how this point can be realised in reality; 17 signatories representing various organisations and institutions (however, unfortunately, the representation of the local community was limited to the local government) have thus committed themselves to a jointly developed and agreed Strategic Plan for the period of 2015–2022. The Model Forest concept is dedicated to areas where there is a problem of conflict or misunderstanding between various social groups and aims to jointly address issues affecting the area (Bator et al. 2014). The Promotional Forest Complex in the Białowieża Forest functions in a similar way. Founded in 1994 aims to protect the natural values of forests of three forest districts: Białowieża, Browsk and Hajnówka. Scientific and Social Councils play an important role in the complex management process engaging various stakeholder groups. However, the given examples do not fully meet our assumed conditions of impartiality due to the fact that they are managed by the State Forests National Forest Holding—so by one of the parties to the dispute (Dyrektor Generalny Lasów Państwowych 2018, 2023; Janeczko and Parzych 2007).

The proposed model accounts for the scientific enterprise and its impact on everyday human life. Not only does it assume that the collective good is broader than the individual good, but it also accepts that the importance of the collective good is to protect and enhance the good of everyone. In the spirit of Kitcher’s model, if science seeks to improve the quality of life, to promote what is good, and to realise our needs, then it can be assumed that the goals of science align with the well-being model of science: “Individual preferences should form the basis for our understanding of the personal good that inquiry (among other social institutions) is to promote. In moving from the individual to the measurement of value for the society, we should explicitly limit our discussions to societies that honour certain democratic ideals. Hence my approach to the fundamental question, «What is the collective good that inquiry should promote?» will start from a subjectivist view of individual value (using personal preferences as the basis for an account of a person’s welfare) and will relate the individual good to the collective good within a framework in which democratic ideals are taken for granted” (Kitcher 2001, p. 116). The principle of autonomy is inextricably linked to the principle of reciprocity here.

Differences of opinion and conflicts constitute grounds for continuing the dialogue, not stopping it.

## Conclusions

The environmental conflict in BF is a good illustration of the potential mechanism for scientists to participate as experts in attempts to define the subject matter of conflict, its nature and methods of its resolution. Broadly speaking, the expert function of science may be realised through the generation of professional scientific knowledge, the exchange of information between scientists, discussion that takes into account the different perspectives of scientific disciplines, and the communication of the fruits of the scientific debate to the wider community (the participants in the conflict). Within the preliminary diagnosis of the environmental conflict, which is largely shared by the ‘scientific’ parties to the dispute, it may be argued that the mechanisms of scientific and social discussion have failed, with the result that the conflict has only been frozen by a judicial verdict of a European institution rather than permanently resolved. The proposed resolution of the conflict in the light of the model of ideal deliberation was intended to shed new light on the above diagnosis: the model of the ideal deliberation implicitly indicates which elements of the actual discussion around the BF protection strategy were inappropriate and what needs to be done so that a potential consensus could be effectively reached. We can mention the following: (1) During the period analysed, the factual dispute over the BF took place through a variety of channels, usually supporting specific ways of protecting the forest. There was no common ground for discussion. The model proposed makes possible gathering representatives of all points of view, not only in the scientific dimension. (2) The factual discourse was limited to BF protection strategies and the dichotomous division of dispute participants. The model assumes an expanded understanding of the problem based on multiple scientific disciplines (expert panel) and the needs of the socio-economic environment. Scientists are not only parties to the conflict, but also experts in the conflict. Deliberation reveals stakeholders’ goals and values. (3) The dispute came down to a polarisation of positions and, as a result, attempts to resolve the dispute in favour of one of them. In the model, success is not about choosing one of the available options, but about arriving at a solution that at least satisfies all stakeholders. Controversial issues of policy and forest management should be guided by the Latin maxim: “quidquid agis, prudenter agas et respice finem”, i.e. “whatever you do, do it wisely and consider what the end will be”.

Our proposal has certain limitations (cf. Rolin 2021). For one thing, the implementation of what has been developed in the course of an ideal deliberation is not solely dependent on the participants. It is possible that the scientific and social consensus that has been developed may be ‘wasted’ by political decision-makers. Secondly, expert scientists may play a key role in the ideal deliberation provided there is a high degree of public trust in science. In an era of pluralistic information sources, high efficiency in propagating false information as well as economic, ideological, political entanglement of science, a high degree of trust in the exclusive relevance of scientific knowledge is problematic. Lack of trust can generate attitudes that question the central importance of this form of knowledge and its creators. Thirdly, the dispute over how to protect the Białowieża Forest has been going on for a very long time, which makes it difficult for discussion participants to reject their prejudices and be open to compromise. Nevertheless, we hope this participatory way of argumentation based on scientific data (the role of scientists), as well as mutual expressing of needs, values and understanding (the general public) will be more involved in the processes of environmental decision making in order to make them truly dialogical and fruitful.

## Footnotes

1 The issue of the participation of various social groups in environmental decisions has not been finally resolved - in 2018, several NGOs complained to United Natons Economic Commission for Europe (UNECE) about the failure of Polish courts to comply with the Aarhus Convention, ratified by Poland in 2003. The complaint concerned the lack of the right to defend nature in courts in the field of activities affecting the environment, and therefore also the health and life of citizens. During the hearing of the parties, the situation regarding the Białowieża Forest was mentioned. The cases are still pending (The United Nations Economic Commission for Europe, 2023).

2 The composition of the Polish government delegation was specified in CJEU judgment, see (Judgment of the Court Grand Chamber, 2018).
